# Does asymmetry in patient recruitment in large critical care trials follow the Pareto principle?

**DOI:** 10.1186/s13063-020-04279-1

**Published:** 2020-05-05

**Authors:** Mahesh Ramanan, Laurent Billot, Dorrilyn Rajbhandari, John Myburgh, Simon Finfer, Rinaldo Bellomo, Balasubramanian Venkatesh

**Affiliations:** 1 Intensive Care Unit, Caboolture and The Prince Charles Hospitals, Brisbane, Australia; 2 University of Queensland, Brisbane, Australia; 3 Critical Care Division, The George Institute for Global Health, Sydney, Australia; 4 Statistics Division, The George Institute for Global Health, Sydney, Australia; 5 University of New South Wales, Sydney, Australia; 6 University of Sydney, Sydney, Australia; 7 Intensive Care Unit, Sydney Adventist Hospital, Sydney, Australia; 8 Intensive Care Unit, Austin Hospital, Melbourne, Australia; 9 University of Melbourne, Melbourne, Australia; 10 Australia New Zealand Intensive Care Research Centre, Melbourne, Australia; 11 Intensive Care Unit, Wesley Hospital, Brisbane, Australia; 12 Intensive Care Unit, Princess Alexandra Hospital, Brisbane, Australia

**Keywords:** Critical illness, Clinical trials, Intensive care unit, Pareto, Trial recruitment

## Abstract

**Background:**

Randomised controlled trials (RCT) may be hindered by slow recruitment rates, particularly in critically ill patients. While statistical models to predict recruitment rates have been described, no systematic assessment has been conducted of the distribution of recruitment across sites, temporal trends in site participation and impact of competing trials on patient recruitment.

**Methods:**

We used recruitment and screening logs from the SAFE, NICE-SUGAR, RENAL, CHEST and ADRENAL trials, five of the largest critical care RCTs. We quantified the extent of recruitment asymmetry between sites using Lorenz curves and Gini coefficients and assessed whether the recruitment distribution across sites follow the Pareto principle, which states that 80% of effects come from 20% of causes. Peak recruitment rates and growth in participating sites were calculated.

**Results:**

In total, 25,412 patients were randomised in 99 intensive care units (ICUs) for the five trials. Distribution of recruitment was asymmetric, with a small number of ICUs recruiting a large proportion of the patients. The Gini coefficients ranged from 0.14 to 0.52. The time to peak recruitment rate ranged from 7 to 41 months and was variable (7, 31, 41, 10 and 40 months). Over time, the proportion of recruitment at non-tertiary ICUs increased from 15% to 34%.

**Conclusions:**

There is asymmetry of recruitment with a small proportion of ICUs recruiting a large proportion of patients. The distributions of recruitment were not consistent with the Pareto principle. There has been increasing participation of non-tertiary ICUs in clinical trials.

## Background

Randomised controlled trials (RCT) in critical care have evolved from small single-centre studies in past decades to large, multicentre, multinational trials with complex methodologies. A key to the successful completion of clinical trials is the recruitment of participants in a timely manner. Recruitment of patients into trials is a major hurdle when performing large RCTs, in critical care and other disciplines [1]. Improving recruitment has been identified as an important priority for improving healthcare research by the National Institute of Health Research [2, 3].

Recruitment patterns into clinical trials have been the subject of multiple investigations, particularly in the areas of oncology [4] and heart failure [5, 6]. Hospital site characteristics may be a critical determinant of enrolment into multicentre trials. Evidence from multicentre heart failure trials has shown that slow enrolment rates have remained unchanged over a 16-year period. Statistical models aimed at predicting recruitment rates and guiding adaptive adjustments in patient recruitment in pharmaceutical trials have been described [7, 8]. Furthermore, these models have been refined to predict recruitment rates at multiple levels including trial-level, region-level and site-level recruitment [8, 9].

In oncology trials conducted in North America, academic centres have been reported to enrol more patients than rural and regional centres, and most of the patient recruitment occurs from a small number of sites. In these trials, the pattern of recruitment reflected the Pareto principle or the “80/20” rule [10, 11], which states that 80% of the effects arise from 20% of the causes. This principle was first described by an Italian economist, Vilfredo Pareto, who observed that 80% of the land in Italy was owned by 20% of the population and later noted that the same was applicable to other phenomena [12–14]. Based on Pareto’s initial findings, a formal mathematical Pareto distribution has been described [12]. Predominately used in economics, the Pareto principle provides a mechanism to evaluate distribution of recruitment into RCTs.

In the last two decades, an increasing number of multi-centre randomised controlled trials have been reported in the intensive care literature, but data on enrolment rates, recruitment patterns and relative contribution of sites are lacking. Owing to the availability of resources, case-mix and capacity, the conduct of and participation in RCTs have largely been limited to tertiary intensive care units (ICU). In the last decade, participation by non-tertiary ICUs in clinical trials has been increasing. Whether this has affected the distribution of recruitment within RCTs is unknown.

The principal objective of this study was to use the Pareto principle to analyse the distribution of recruitment into multicentre, critical care RCTs. Our primary hypothesis was that there would be asymmetry in the distribution of recruitment across sites within each trial, with a small number of sites accounting for a large proportion of recruitment. Our secondary hypotheses were that the increased participation of non-tertiary sites over time will result in less asymmetric recruitment distribution and that increasing the number of concurrent trials in which an individual ICU was participating will reduce the rate of recruitment into individual trials.

Using data from five large-scale investigator-initiated RCTs in critical care that were conducted over two decades, we performed this study to evaluate whether the distribution of recruitment across sites follows the Pareto principle. We also assessed the extent of participation of non-tertiary sites into clinical trials over time and the impact of concurrent trials on the rate of recruitment.

## Methods

We performed a retrospective analysis of recruitment logs from five multicentre RCTs conducted through the Australia New Zealand Intensive Care Society (ANZICS) Clinical Trials Group network (the Clinical Trials Group) and co-ordinated by The George Institute for Global Health (The George Institute).

The Clinical Trials Group is a network of critical care researchers who design and conduct multi-centre, investigator-initiated, collaborative research in Australia and New Zealand ICUs with participation from international partners.

### Data sources

Recruitment and feasibility logs were sourced from The George’s Institute data repository. These contained the date and site of randomisation. The recruitment time periods and full titles for the five trials, SAFE [15], NICE-SUGAR [16], RENAL [17], CHEST [18] and ADRENAL [19], are listed in Table 1.

**Table 1:** This is a table showing characteristics of each trial including recruitment period, participating sites and average recruitment rates

	SAFE	NICE-SUGAR	RENAL	CHEST	ADRENAL
Full Title	A comparison of albumin and saline for fluid resuscitation in the intensive care unit	Intensive versus conventional glucose control in critically ill patients	Intensity of continuous renal-replacement therapy in critically ill patients	Hydroxyethyl starch or saline for fluid resuscitation in intensive care	Adjunctive glucocorticoid therapy in patients with septic shock
Recruitment period	November 2001 to June 2003	December 2004 to November 2008	December 2005 to August 2008	December 2009 to January 2012	March 2013 to April 2017
Number of patients	7000	6104	1508	7000	3800
Number of participating ICUs	16	42	35	32	69
Tertiary ANZ^a^ ICUs n (%)	13 (81)	15 (36)	22 (63)	16 (50)	24 (35)
Non-tertiary ANZ ICUs n (%)	3 (19)	10 (24)	13 (37)	16 (50)	29 (42)
Non-ANZ ICUs n (%)	0 (0)	17 (40)	0 (0)	0 (0)	16 (23)
Number of patients recruited in tertiary ANZ ICUs n (%)	5917 (85)	4118 (67)	1179 (78)	5246 (75)	1859 (49)
Number of patients recruited in non-tertiary ANZ ICUs n (%)	1083 (15)	1230 (20)	329 (22)	1754 (25)	1280 (34)
Number of patients recruited in non-ANZ ICUs n (%)	0 (0)	756 (12)	0 (0)	0 (0)	661 (17)
Months of recruitment	18.8	44.4	32.1	25.2	58.3
Peak daily recruitment rate	18.42	5.65	2.36	14.89	3.3
Peak monthly recruitment rate	560.3	171.9	71.8	452.9	100.4
Average daily recruitment rate	12.26	4.52	1.55	9.11	2.14
Average monthly recruitment rate	372.9	137.5	47.1	277.1	65.1
Number of concomitant ANZICS^b^ CTG^c^ trials	0	4	4	9	13
Total patients recruited per site (Mean (SD))	438 (130.7)	145 (155.5)	43 (44.9)	219 (192.1)	55 (48.6)
Median (IQR)	444 (330–524)	97 (40–221)	29 (19–46)	156 (75–315)	22 (38–72)
Range	225–656	3–647	6–180	2–767	3–264
Monthly recruitment per site (Mean (SD))	28.9 (6.96)	4.5 (3.6)	1.7 (1.6)	11.4 (9)	1.4 (1)
Median (IQR)	28 (23–35)	3.4 (1.6–5.7)	1.4 (0.8–1.8)	8.8 (4.5–16)	1 (0.7–1.8)
Range	18–41	0.7–17	0.4–6.8	0.3–31	0.1–5

^a^ ANZ- Australia and New Zealand

^b^ ANZICS- Australia New Zealand Intensive Care Society

^c^ CTG- Clinical Trials Group

We determined the tertiary or non-tertiary status of individual ICUs in Australia and New Zealand (ANZ) based on their level of accreditation by the College of Intensive Care Medicine [20], which is the training body for intensive care medicine in Australia and New Zealand. A tertiary ICU in the ANZ setting denotes a referral ICU, which is capable of providing comprehensive multi-organ life support for an indefinite time period and has a demonstrated commitment to academic education and research. ICUs from outside ANZ were not categorised into tertiary and non-tertiary due to international variations in definitions.

Data access from The George Institute and College of Intensive Care Medicine was approved by the responsible authorities within each organization. All data were extracted and stored in encrypted Microsoft Excel spreadsheets. The recruitment logs contained the recruitment numbers, dates and sites (ICUs) for each RCT.

### Statistical analyses

Recruitment sites were categorised as either tertiary, non-tertiary or other (i.e., from outside Australia and New Zealand). The number of sites in each category and the number and proportion of patients recruited at each of these were calculated for each trial to determine whether the proportion of patients recruited at non-tertiary sites increased over time from 2001 to 2017.

For a quantitative analysis of the distribution of recruitment across the sites within a trial and to study the Pareto principle in the context of RCTs, we applied the Gini coefficient and the Lorenz curve, techniques developed in the early Twentieth Century and widely used in economics to study the inequalities of wealth and income distribution [21, 22]. Briefly, the Lorenz curve plots the proportion of the population, in our case the recruiting ICUs or sites, on the x-axis, versus the cumulative income distribution, in our case the recruitment, (Additional File 1: Derivation of Lorenz curve). A straight line running from co-ordinates (0,0) to (1) represents perfect equality; that is, each site recruits exactly the same proportion of patients. Deviation from this line is proportional to inequality; that is, the further the actual Lorenz curve is from this diagonal line, the greater the proportion of recruitment that occurs at a small number of sites. Mathematically, the Gini coefficient (range 0 to 1) can be calculated to quantify the degree of inequality. The diagonal line, representing perfect equality, has a Gini coefficient of 0. The higher the Gini coefficient, the more unequal or asymmetric the distribution. We applied this to our analysis by ordering highest to lowest recruiting sites on the x-axis and the corresponding proportion of cumulative recruitment on the y-axis. The area above the Lorenz curve was calculated and used to derive the Gini coefficient. We adjusted [23, 24] for the amount of time for which each site recruited (not all sites start recruiting at the same time) and, where available, for the annual number of admissions at each site. Recruitment asymmetry has previously been quantified using Poisson-gamma models [7]. Pharmaceutical trial data have been used to demonstrate that the results of Poisson-gamma modelling, when used to demonstrate recruitment asymmetry, closely mirror the results obtained from constructing Lorenz curves [8].

The SAFE trial was the first major multicentre trial conducted by the Clinical Trials Group. Sixteen sites participated in the SAFE trial, 14 of which were tertiary sites. Many of these sites participated in multiple trials conducted by the Clinical Trials Group. To assess the relative and ongoing contribution of these sites to the trials, which had a relatively constant presence across the five trials, we compared the proportion of recruitment in each trial that occurred at the SAFE trial sites. Monthly recruitment rates at each of these sites within each trial were plotted using restricted cubic spline smoothing.

The number of concurrently running Clinical Trials Group trials that were recruiting patients in Australia and New Zealand ICUs at the same time as each of the five trials were identified.

All analyses were performed in Stata 13.0 or Microsoft Excel.

## Results

The trial methodology and the full results from the five trials have been reported in detail elsewhere. The trials recruited a total of 25,412 patients from 99 ICUs, mainly in Australia and New Zealand (ANZ) but also from Canada, the United Kingdom, Ireland, Saudi Arabia and Denmark. As the five trials span different time periods with minimal overlap, they are representative of changes over nearly two decades in critical care trials in Australia and New Zealand. The mean and peak daily and monthly recruitment rates, along with other trial level data, are presented in Table 1.

### Pareto analysis: distribution of recruitment

**Fig. 1:**
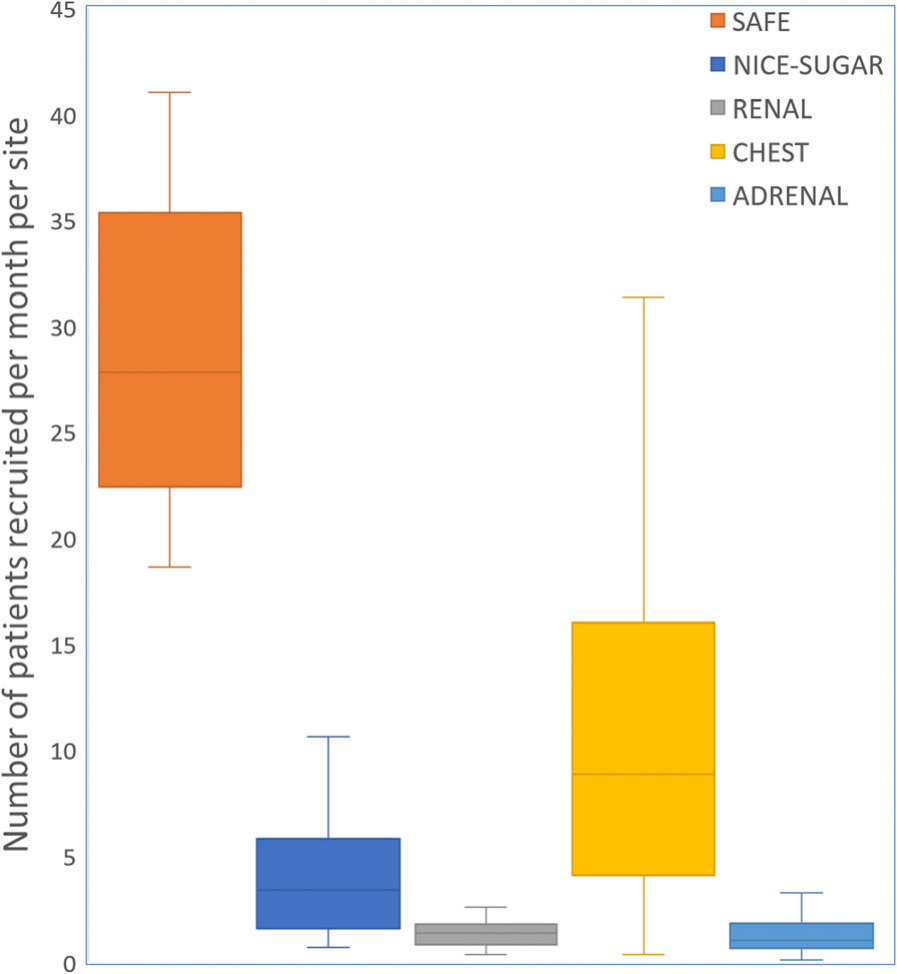
Boxplots of monthly recruitment rates. Box-and-whiskers plots depicting median monthly recruitment rate per site by trial

**Fig. 2:**
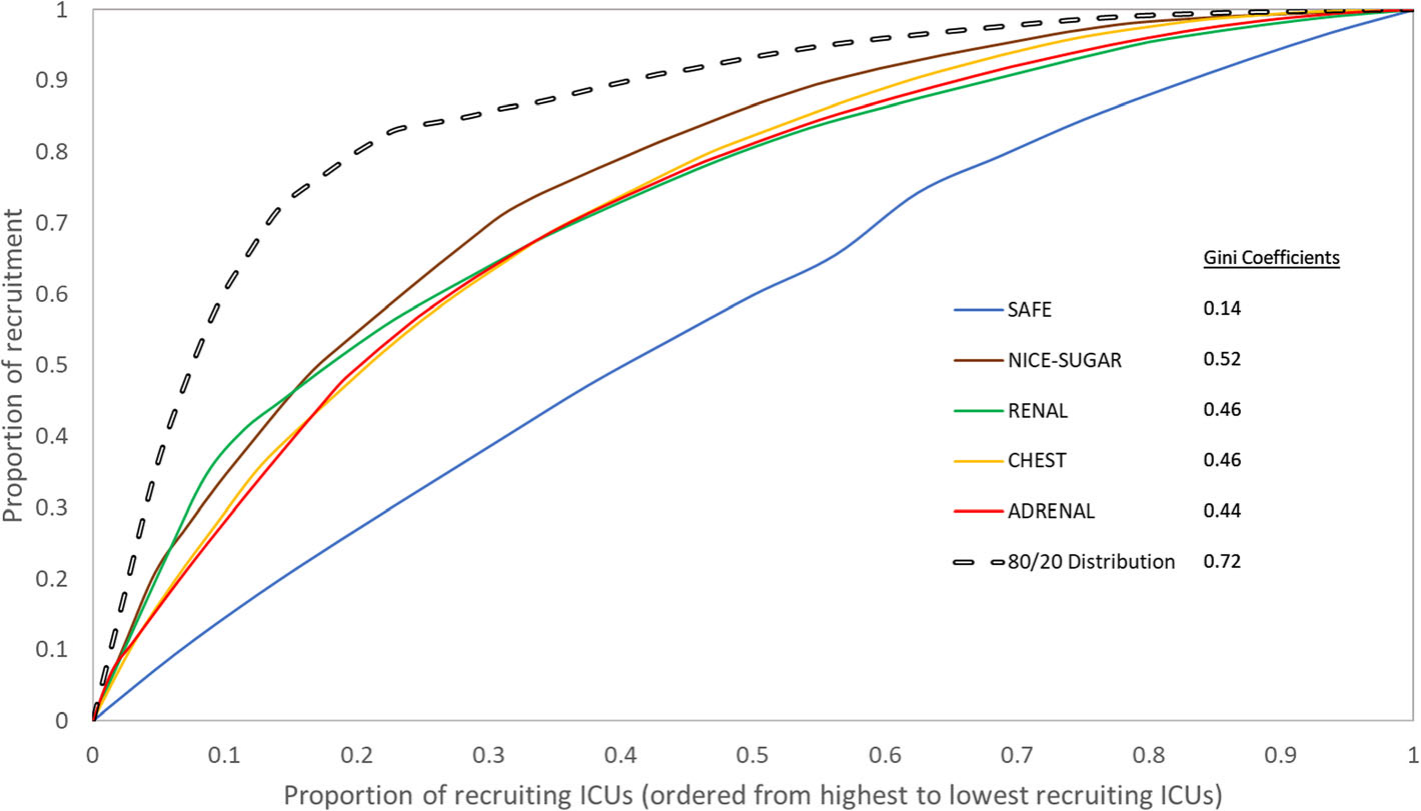
Lorenz curves. Lorenz curves and Gini coefficients for the five trials. The grey diagonal line is the line of equality, which represents a scenario where each ICU recruits the same proportion of patients into the trial. Each coloured line is a Lorenz curve for a trial, with greater distance from the line of equality representing greater asymmetry of recruitment. The dashed line is the “80/20” distribution where 80% of the recruitment occurs in the top 20% of sites

The distribution of monthly recruitment rates per site by trial is shown in Fig. 1. SAFE and CHEST both had high recruitment rates with median monthly recruitment of 28 (IQR 23–35) and 8.8 (4.5–16) per site respectively. The other three trials had median rates between 1 and 3.4. Inspection of the Lorenz curves (Fig. 2) revealed asymmetry of recruitment; however, the Pareto principle was not observed in any of the five trials. In total, 80% of the patients were recruited by the highest recruiting 41% to 70% of ICUs rather than 20%. SAFE had the least asymmetry in the distribution of recruitment with a Gini coefficient of 0.14, probably because it involved a relatively small number of mainly tertiary ICUs. The Lorenz curves and Gini coefficients of the remaining four trials were clustered together (Fig. 2). NICE-SUGAR had a Gini coefficient of 0.52, RENAL and CHEST 0.46 and ADRENAL 0.44. After adjusting for annual number of admissions for each site for CHEST and ADRENAL (data not available for the other trials), the Gini coefficient remained 0.44 for ADRENAL and dropped slightly from 0.46 to 0.44 for CHEST.

### Recruitment by ICU – tertiary versus non-tertiary

**Fig. 3:**
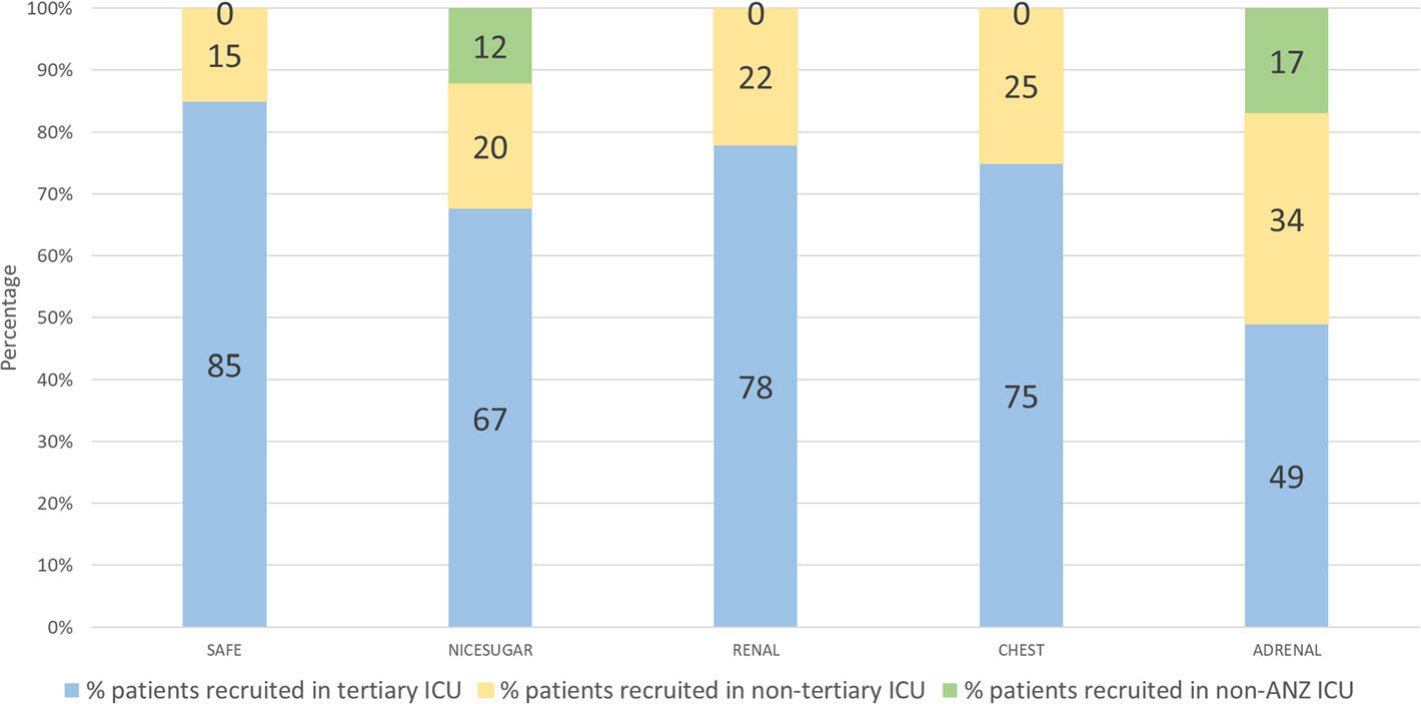
Proportion of recruitment in different ICUs**.** Proportion of recruitment in tertiary, non-tertiary and non-ANZ ICUs in each of the five trials. *ANZ* Australia and New Zealand

The temporal changes in numbers of different types of ICUs, tertiary, non-tertiary and other, and patients recruited in each of these types of ICUs from SAFE to ADRENAL are presented in Table 1 and Fig. 3. Overall, there was an increase in the number of participating ICUs from 16 in SAFE to 69 in ADRENAL, with a substantial increase in the proportion of non-tertiary ICUs (19% in SAFE to 42% in ADRENAL). Correspondingly, the proportion of patients in each trial recruited by non-tertiary ICUs increased from 15% in SAFE to 20–25% in NICE-SUGAR, RENAL and CHEST to 34% in ADRENAL (Fig. 3). Two of the trials had non-ANZ ICU participation, with 17 in NICE-SUGAR and 16 in ADRENAL.

### Recruitment at SAFE sites that participated in subsequent trials

SAFE sites accounted for 22 to 40% of total sites in the subsequent four trials. They accounted for 38 to 50% of the total recruitment (Additional File 1: Supplementary Table 1). A decline over time occurred in the proportion of total recruitment that occurred at these sites. Some variability existed in when the sites reached their peak recruitment rate within each trial (Additional File 1: Supplementary Figures 1–5). In SAFE, NICE-SUGAR and CHEST, most sites reached the peak recruitment rate early in the trial, with a subsequent decline at some sites. In RENAL and ADRENAL, a spread of sites reached peak recruitment rate in the early, middle and late stages of recruitment.

### Impact of concurrent trials on trial duration

The number of concomitantly recruiting ANZICS Clinical Trials Group trials increased from zero for SAFE to four each for NICE-SUGAR and RENAL (which had extensive overlap in their recruitment periods), nine for CHEST and 13 for ADRENAL (Table 1); however, no direct effect of the number of concurrent trials on the trial recruitment rate was apparent, with CHEST reporting much higher recruitment rates than ADRENAL despite a large number of concurrent trials.

## Discussion

### Key findings

We have described a novel use of the Pareto principle by applying the Lorenz curve and Gini coefficient to analyse the distribution of patient recruitment into clinical trials. Asymmetry of recruitment occurred, with a relatively small proportion of ICUs recruiting a large proportion of patients in the critical care RCTs; however, the degree of asymmetry was not in keeping with what would be expected under the Pareto principle. Substantial growth occurred in the participation and recruitment by non-tertiary ICUs with time. The effect of concurrently recruiting trials on recruitment rates could not be adequately studied in our sample of trials.

### Importance of findings

Resources for conducting high-quality critical care RCTs have traditionally been concentrated in large, academic, university-affiliated, tertiary ICUs. However, with the increasing number and size of RCTs, expanding the pool of recruiting sites has become necessary to conduct timely, efficient research that is also broadly applicable. In our study, we have shown a temporal increase in the number of and recruitment from non-tertiary ICUs.

Distribution analysis using Lorenz curves and Gini coefficients showed a small number of sites recruiting a high proportion of patients after adjustment for recruitment time and volume. This potentially indicates that a strategy of solely focussing on the high recruiting sites, which are located at the left of the Lorenz curve, is unlikely to yield improvements in the overall recruitment rate. Instead, gains in recruitment speed could be made by focussing on sites at the middle and right of the Lorenz curve. Possible mechanisms for this include allowing triallists to target interventions aimed at boosting recruitment rates (such as education, site visits and reallocation of funds) at slow recruiting sites, closing slow recruiting sites and better selecting sites when designing a trial. The impact of faster recruitment could include a reduction in project management costs and drug/device costs (less likely to expire) and faster dissemination of potentially practice-altering results.

We propose that the Gini coefficient could be used as a marker of external validity of clinical trials. A high Gini coefficient, due to asymmetry in recruitment distribution, may be associated with a reduction in external validity and hence limitations in the generalisability of trial results. A high Gini coefficient (after adjustment for site size), for example, would indicate that a large proportion of recruitment occurred in a small number of sites. Therefore, even if a trial had a large number and variety of participating sites, in this situation, the results may not be broadly generalizable. A high Gini coefficient, therefore, could point to limited external validity and generalizability of trial results. Conversely, a low Gini coefficient or a strong departure from asymmetry may indicate higher external validity. The Gini coefficient could also be used to compare the external validity of two or more trials evaluating a similar question.

Our results have several implications for researchers conducting critical care RCTs globally. Serial examination of the Lorenz curve and Gini coefficient, for example at 25% and 50% of enrolment, could be used as a quality marker during the conduct of RCTs, for assessing external validity and for comparing recruitment between RCTs. When planning an RCT, the historically highest recruiting sites (those that occupy the left-hand end of the Lorenz curve) in past trials or in pilot trials, could be identified and targeted first; however, some caution should be exercised as site performance can vary from trial to trial [25]. This would aid trials groups and co-ordination centres in site selection. Funding bodies may also find it more desirable to fund groups that have a record of performing trials with low levels of recruitment asymmetry and high external validity.

However, with the increasing numbers of RCTs being conducted and potentially competing for patients, strategies to enable more sites to participate in RCTs are necessary. Particular site-level characteristics likely exist that enable sites to be high recruiters. Further research should focus on identifying these site-level characteristics, which are potentially related to research infrastructure, and applying these to other sites to help those sites increase their recruitment rates. One way to do this would be to prospectively embed an analysis like ours into an RCT [26] with a view to investigating the effect of specific site-level characteristics on the distribution of recruitment. This would have implications, not just for researchers and funding bodies but also for healthcare policymakers in building healthcare systems that have greater research capacity. Systematic differences may occur in the distribution of recruitment into cluster-randomised trials, commercial or industry-sponsored trials and trials conducted in other regions, in comparison to investigator-initiated individual patient trials conducted in ANZ, as we have described in this manuscript. A comparative distribution analysis of a broad variety of trials may be useful in determining whether such systematic differences exist.

### Strengths and limitations

The strengths of our study are that it used a large high-quality dataset and incorporated site-level data. It is the first attempt, to our knowledge, of analysing recruitment patterns in critical care RCTs and the first use of Lorenz curves and Gini coefficients to display and study the distribution of recruitment within RCTs. Although statistical models that incorporate site-level variability and predict trial enrolment have been described in a review article by Heitjan, Ge and Ying [27], our broad literature search failed to find any previously published analysis of recruitment patterns and distribution that were comparable to this study.

Our study had some limitations. It is a retrospective analysis of data, and not all relevant data points were available, particularly site-level information pertaining to research infrastructure, which is a likely confounder in an analysis of recruitment into RCTs. The five trials, despite having some similarities, have major differences in terms of the patient population, the interventions and the time period in which they were conducted. Heterogeneity likely exists between the characteristics for which these trials have not been adjusted. The study was predominantly confined to sites in Australia and New Zealand that have different research infrastructure set-up and resource allocation as compared to other geographic regions.

## Conclusions

In conclusion, we have shown that asymmetric distribution of recruitment occurs in critical care RCTs by applying the Pareto principle. We have described a novel use of the Lorenz curve and Gini coefficient, which can be used to generate easily understood metrics to quantify asymmetry. This approach may inform triallists about site selection and trial management, assist in evaluating external validity and be used by healthcare policymakers to build healthcare systems that have greater research capacity.

## Supplementary information

**Additional file 1.**

## Data Availability

The datasets used in the current study are available from the corresponding author on request. The George Institute for Global Health Data Sharing Policy (found at the following URL https://www.georgeinstitute.org/data-sharing-policy) will be applicable.

## Abbreviations

RCT: randomised controlled trial
ICU: intensive care unit
ANZICS: Australia New Zealand Intensive Care Society
SAFE: a comparison of albumin and saline for fluid resuscitation in the intensive care unit
NICE-SUGAR: intensive versus conventional glucose control in critically ill patients
RENAL: intensity of continuous renal-replacement therapy in critically ill patients
CHEST: hydroxyethyl starch or saline for fluid resuscitation in intensive care
ADRENAL: adjunctive glucocorticoid therapy in patients with septic shock
ANZ: Australia and New Zealand
