# Supplementary material for: Does asymmetry in patient recruitment in large critical care trials follow the Pareto principle?
Source: Trials. 2020 May 5;21:378. doi: 10.1186/s13063-020-04279-1 (PMC7201735; doi:10.1186/s13063-020-04279-1)
Supplement: Supplementary file 1 — Additional file 1. [file 13063_2020_4279_MOESM1_ESM.pdf]

Electronic Supplementary Material

Table of contents

Derivation of Lorenz curve……………………………………………………………………………………………………..Page 2

Explanatory note: Recruitment at SAFE trials sites in subsequent trials…………………………………….Page 3

Supplementary Figure 1: Monthly recruitment- SAFE…………………………………………………………………Page 4

Supplementary Figure 2: Monthly recruitment- NICE-SUGAR…………………………………………………….Page 5

Supplementary Figure 3: Monthly recruitment- RENAL………………………………………………………………Page 6

Supplementary Figure 4: Monthly recruitment- CHEST……………………………………………………………….Page 7

Supplementary Figure 5: Monthly recruitment- ADRENAL…………………………………………………………...Page 8

Supplementary Figure 6: Monthly recruitment rates in the five trials……………………………………...Page 9

Supplementary Table 1…….Page 10

Title: Analysing recruitment into randomised trials using the Pareto distribution

Journal Name: Intensive Care Medicine

Authors: M Ramanan, D Rajbhandari, J Myburgh, L Billot, S Finfer, R Bellomo, and B Venkatesh

Corresponding author: M Ramanan

Staff Specialist, Caboolture Hospital, QLD, Australia

Email: Mahesh.ramanan@health.qld.gov.au

Derivation of Lorenz curve

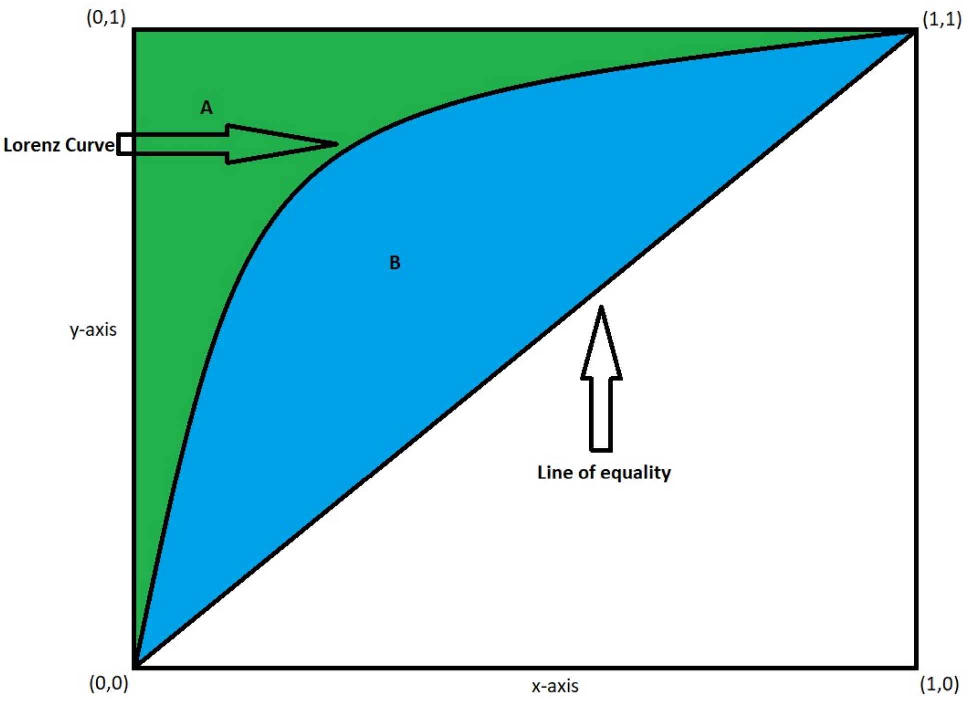

Gini coefficient = B/(A+B)

x-axis: cumulative proportion of "causes" (i.e. sites) ordered from those with the highest proportion of "effects" (i.e. recruitment) to those with the lowest proportion

y-axis: cumulative proportion of "effects" (i.e. recruitment)

Explanatory note: Recruitment at SAFE trial sites in SAFE and subsequent trials

The SAFE trial was the first major, multicentre trial conducted by the ANZICS CTG. There were 16 sites that participated in the SAFE trial, 14 of which were tertiary sites. Many of these sites participated in multiple ANZICS CTG trials including NICE-SUGAR, RENAL, CHEST and ADRENAL. To assess contribution of these sites across the trials, monthly recruitment rates at each of these sites within each trial were plotted using locally weighted scatterplot smoothing (LOWESS). The monthly recruitment at these sites that were common to all five trials are presented in the following pages.

Each trial is represented on a separate figure. There are multiple LOWESS curves per figure corresponding to the number of sites out of the 16 SAFE trial sites that participated in the respective trial. For example, there are 15 curves for ADRENAL as there were 15 out of 16 SAFE trial sites that participated in ADRENAL.

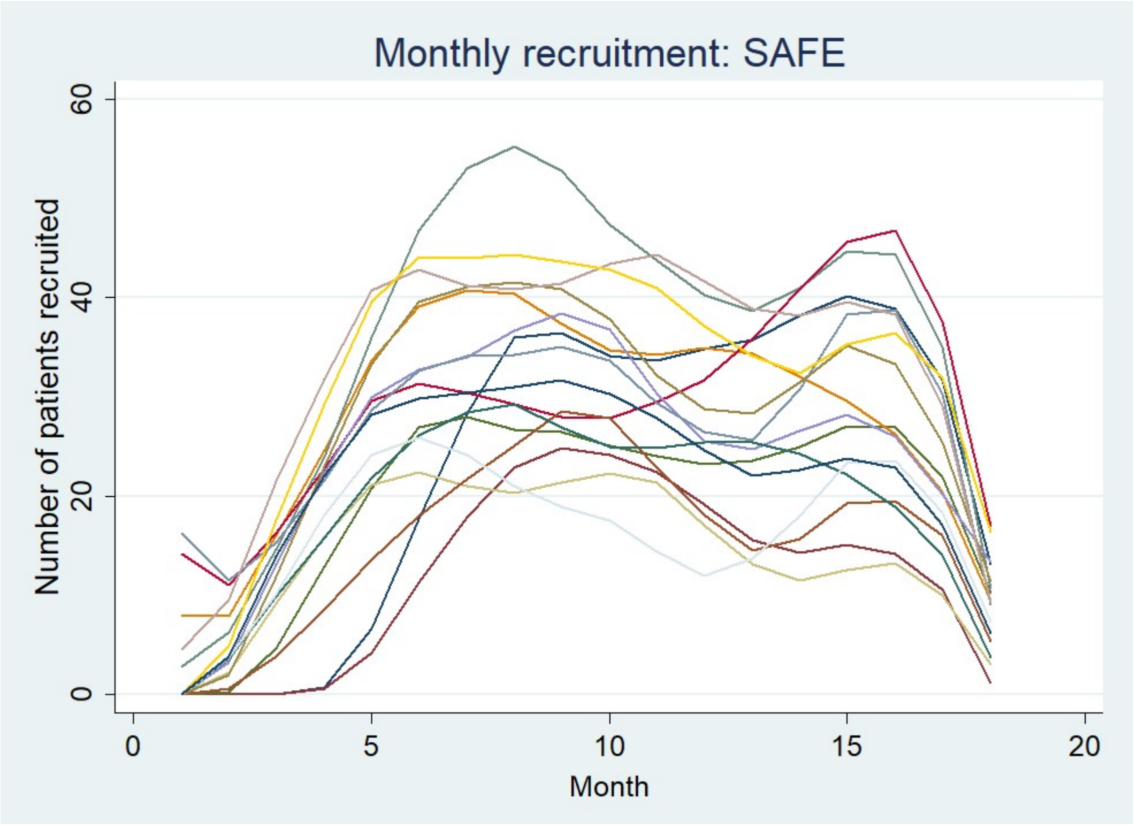
Monthly recruitment: SAFE
Number of patients recruited
0
20
40
60
0
5
10
15
20
Month

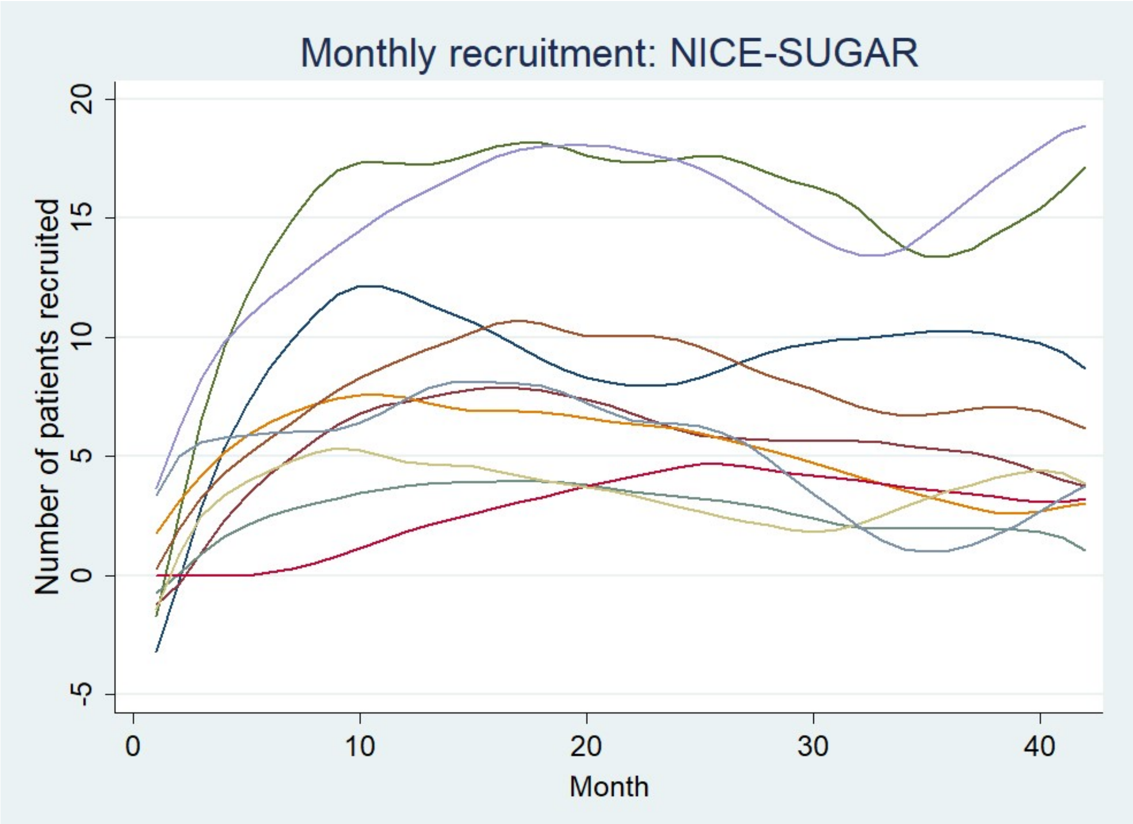
Monthly recruitment: NICE-SUGAR
Number of patients recruited
Month
-5
0
5
10
15
20
0
10
20
30
40

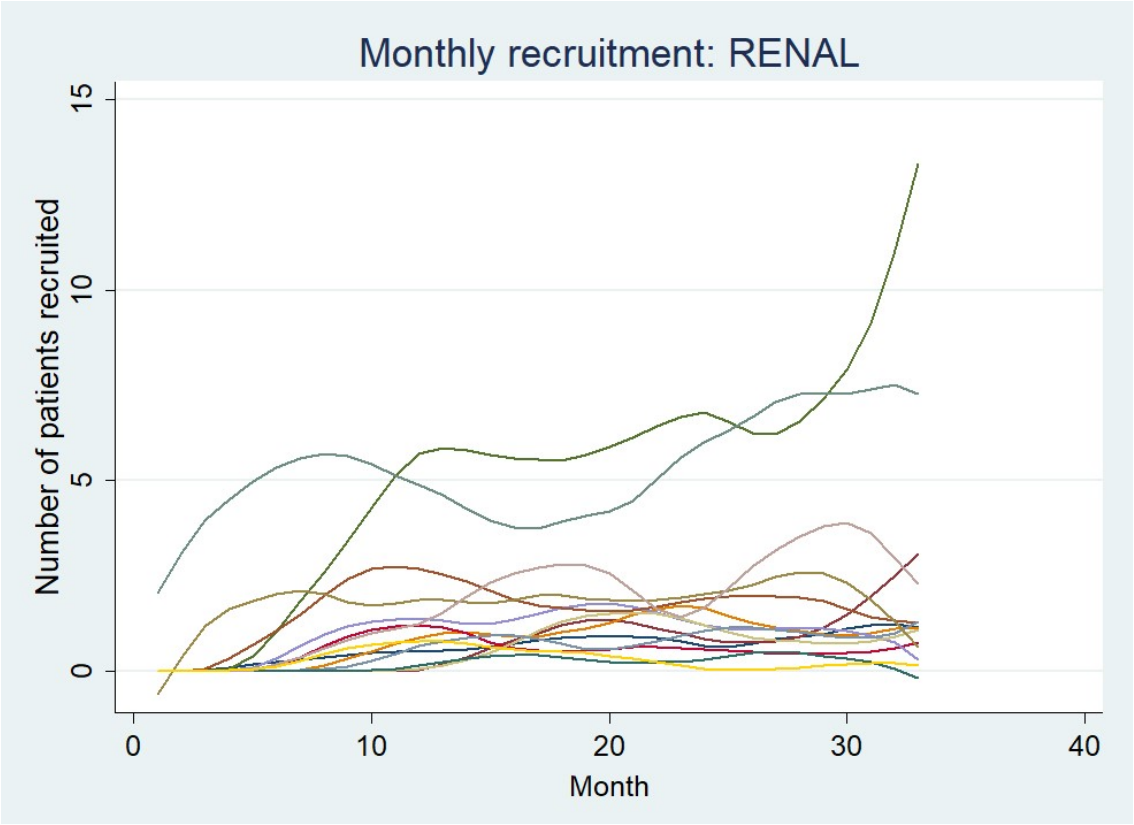
Monthly recruitment: RENAL
Number of patients recruited
0
5
10
15
0
10
20
30
40
Month

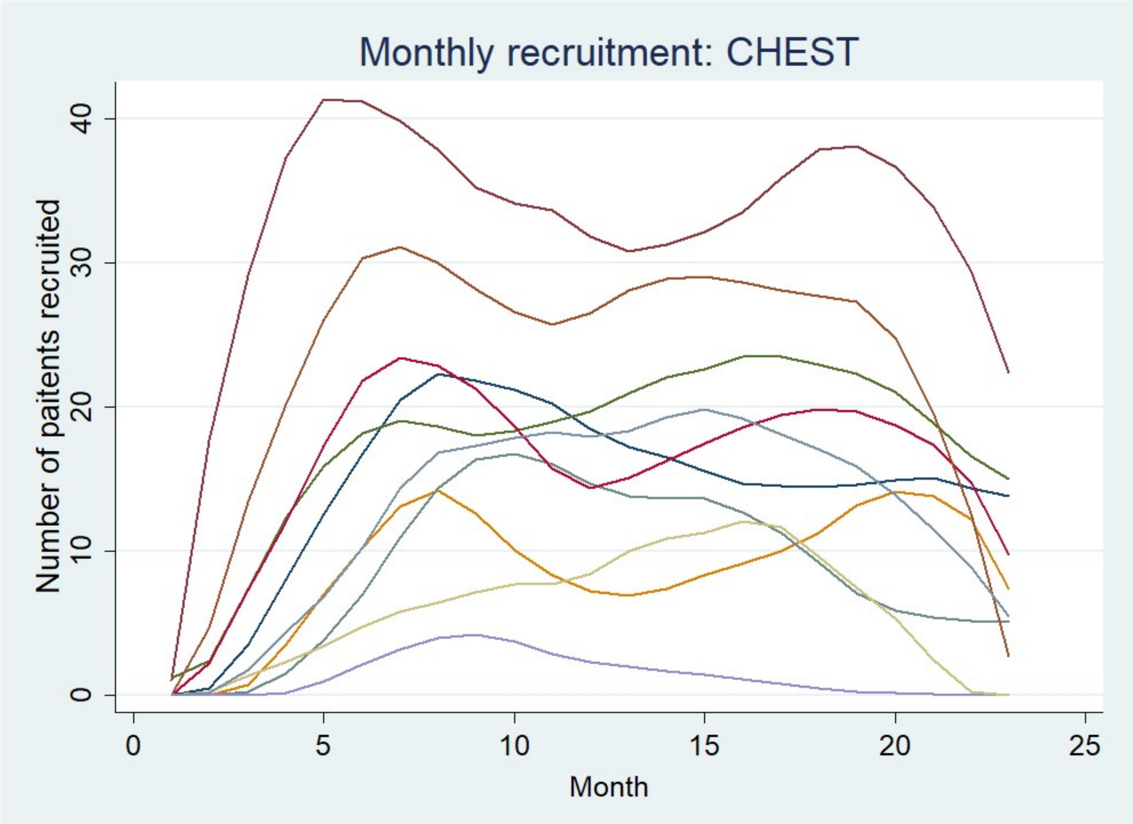
Monthly recruitment: CHEST
Number of paitents recruited
0
10
20
30
40
0
5
10
15
20
25
Month

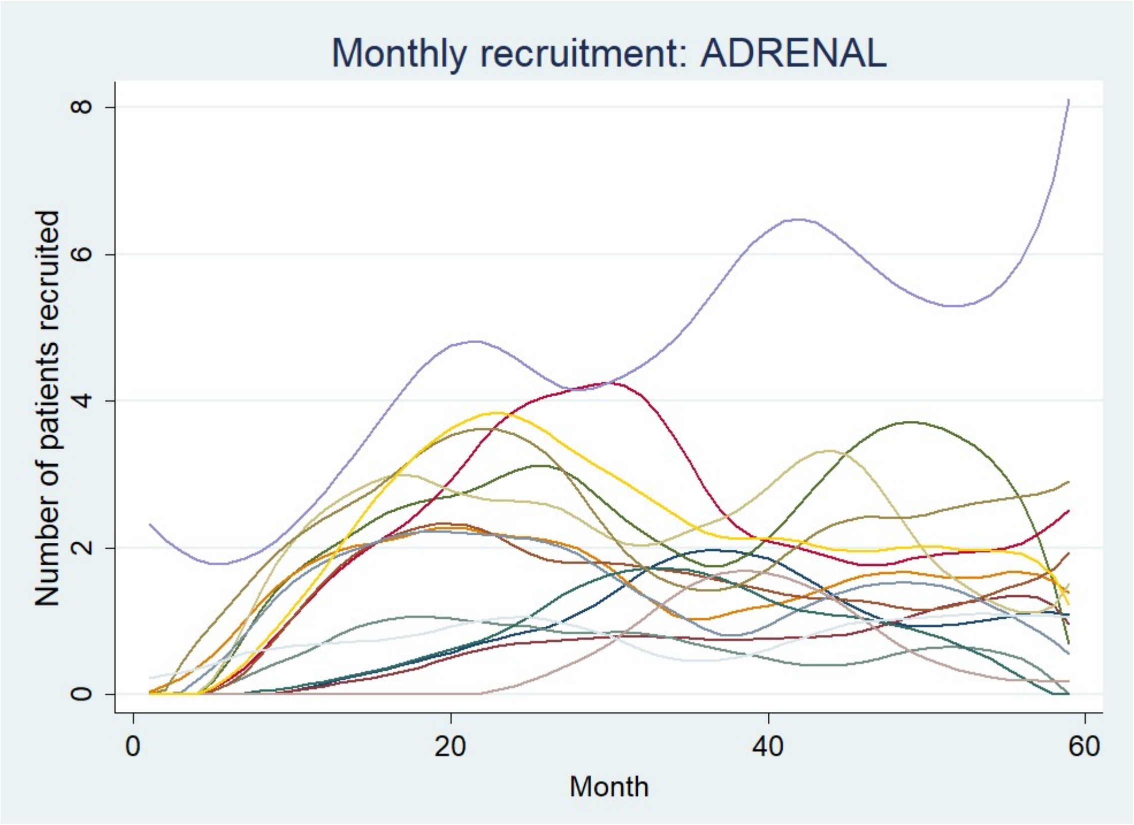
Monthly recruitment: ADRENAL
Number of patients recruited
0
2
4
6
8
0
20
40
60
Month

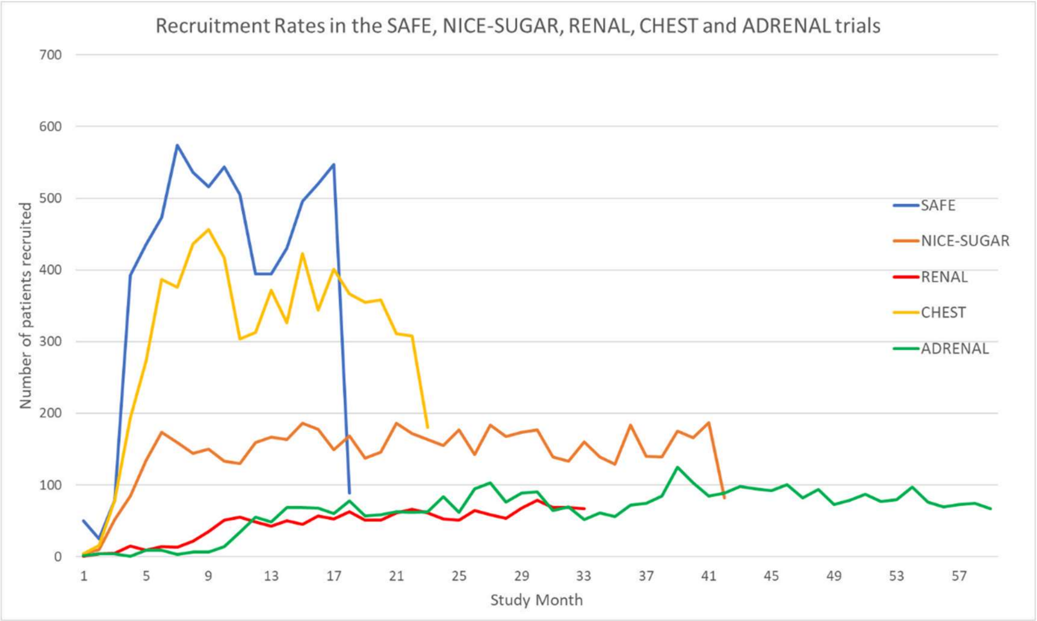
Recruitment Rates in the SAFE, NICE-SUGAR, RENAL, CHEST and ADRENAL trials
Number of patients recruited
Study Month
SAFE
NICE-SUGAR
RENAL
CHEST
ADRENAL
0
100
200
300
400
500
600
700
1
5
9
13
17
21
25
29
33
37
41
45
49
53
57

Supplementary Table 1

Trial	Number of SAFE sites participating	Total number of participating sites	Percentage of sites which participated in SAFE %	Percentage of total recruitment %
SAFE	16	16	100	100
NICE-SUGAR	10	42	24	50
RENAL	14	35	40	46
CHEST	10	32	31	47
ADRENAL	15	69	22	38
